# Genome-Wide Patterns of Homozygosity and Relevant Characterizations on the Population Structure in Piétrain Pigs

**DOI:** 10.3390/genes11050577

**Published:** 2020-05-21

**Authors:** Huiwen Zhan, Saixian Zhang, Kaili Zhang, Xia Peng, Shengsong Xie, Xinyun Li, Shuhong Zhao, Yunlong Ma

**Affiliations:** Key Laboratory of Agricultural Animal Genetics, Breeding, and Reproduction of the Ministry of Education & Key Laboratory of Swine Genetics and Breeding of the Ministry of Agriculture, Huazhong Agricultural University, Wuhan 430070, China; wenhuizhan412@gmail.com (H.Z.); zhangsaixian@163.com (S.Z.); zkl1153793935@163.com (K.Z.); pengxia418@163.com (X.P.); ssxie@sibcb.ac.cn (S.X.); xyli@mai.hzau.edu.cn (X.L.); shzhao@mail.hzau.edu.cn (S.Z.)

**Keywords:** ROH islands, inbreeding coefficients, effective population size, Piétrain pigs

## Abstract

Investigating the patterns of homozygosity, linkage disequilibrium, effective population size and inbreeding coefficients in livestock contributes to our understanding of the genetic diversity and evolutionary history. Here we used Illumina PorcineSNP50 Bead Chip to identify the runs of homozygosity (ROH) and estimate the linkage disequilibrium (LD) across the whole genome, and then predict the effective population size. In addition, we calculated the inbreeding coefficients based on ROH in 305 Piétrain pigs and compared its effect with the other two types of inbreeding coefficients obtained by different calculation methods. A total of 23,434 ROHs were detected, and the average length of ROH per individual was about 507.27 Mb. There was no regularity on how those runs of homozygosity distributed in genome. The comparisons of different categories suggested that the formation of long ROH was probably related with recent inbreeding events. Although the density of genes located in ROH core regions is lower than that in the other genomic regions, most of them are related with Piétrain commercial traits like meat qualities. Overall, the results provide insight into the way in which ROH is produced and the identified ROH core regions can be used to map the genes associated with commercial traits in domestic animals.

## 1. Introduction

The Piétrain pig originated in Belgium. It was founded by crossing local swine with Berkshire, English Large White and Bayeux pig and then was exported to other countries after 1950s. Piétrain pig has medium size, well-developed muscles and white skin with black spots. It is unavoidable to create close inbreeding during breed formation [1]. Whereas those strong inbreeding and selection shaped the Piétrain’s important characteristics like high lean percentages [2]. Currently, this breed is popular around the world, and it is commonly used as terminal sire in the commercial crossing system.

Runs of homozygosity (ROH) are consecutive homozygous segments. The autozygous regions exist due to parents descending the same haplotypes to their progeny [3]. In the process of evolution, multiple factors including mutation, migration, random genetic drift and selection pressure make genotype frequency change, which consequently contributes to change the characterization of runs of homozygosity. In animal genetics, the occurrence of homozygous segments is influenced by the locations of recessive-disease, population history as well as consanguinity levels [4]. The size of effective population (*Ne*) can also reflect the patterns of runs of homozygosity indirectly. As an important parameter of population genetics, it provides an important reference for genome architecture research.

At present, compared with other existing methods, the whole genome inbreeding coefficient based on ROH (*F_ROH_*) is considered to be the most accurate calculation method for predicting the inbreeding level of each individual [5,6]. In addition, the inbreeding coefficient estimated from ROH can be divided into recent and ancestral inbreeding events [5]. Therefore, ROH is applied to predict inbreeding effects in animal genetics [7,8,9]. In theory, the variations productized by an individual would transmit to its offspring, and then they may share identical genomic segments [4]. Therefore, ROH can be used to optimize mating schemes for minimizing inbreeding in livestock breeding [10,11]. In addition, ROH is also used to detect associations between genotype and phenotype, and to identify regions associated with traits of economic interest in farm animals [11,12,13].

In general, ROH patterns in farm animals are aligned with their breeding history. There was a significant correlation between shared short ROH and genomic regions under selection in a population [11]. Accordingly, those ROH regions are probably the results of natural selection and long-term breeding programs. In theory, the genomic regions that were shaped by selection often show a significant reduction in nucleotide diversity and increase in homozygosity around the causal mutation [4]. Therefore, the genomic regions underlying artificial selection in the process of animal breeding are often connected with the high-frequency ROH regions [13]. It suggested that the position of runs of homozygosity may imply the genomic segments that have been subjected to selective pressure [14,15].

Previous ROH studies focused on human and discussed the association between homozygous and complex diseases like stomach cancer, heart diseases, schizophrenia and albinism [16,17,18,19]. The autozygosity of deleterious recessive allele can reflect the cause of recessive-disease effectively [4]. With the development of high-density Chip and sequencing technology, it becomes easier to do homozygous studies. In addition, it benefits from the development of ROH detection methods [20]. Accordingly, researches in the causes and patterns of homozygosity have received more attention in recent years.

The present study is based on the detection of runs of homozygosity, investigating the genomic distribution and features of ROH in Piétrain pig using 50k single nucleotide polymorphism (SNP) GeneChip data. We further investigated the biologic significance of high frequency ROH islands. As expected, we find that ROH islands are associated with some important economic traits, such as meat quality. In addition, the whole genome inbreeding coefficient based on ROH is assessed for prediction ability through comparison with traditional pedigree inbreeding coefficient and other genomic inbreeding coefficients. Overall, the results from this study can provide insights into the pattern of runs of homozygosity and direct its application in animal breeding.

## 2. Materials and Methods

### 2.1. Data Preparation

376 Piétrain pigs that belonged to the same generation without generation overlapping were used. All research involving animals was conducted under protocols (No. 5 proclaim of the Standing Committee of Hubei People’s Congress) approved by the Standing Committee of Hubei People’s Congress and the ethics committee of Huazhong Agricultural University in China. Pigs were genotyped for 50,264 SNPs spread over all chromosomes using Illumina PorcineSNP50 Bead Chip. Only SNPs on autosomes were considered in the following ROH analyses. All analyses were based on *Sus scrofa* (Pig) 11.1 reference genome version. The next step was to quality-control using PLINK software and R programming. The filtering parameters were as follows: (ⅰ) SNPs with a call rate less than 95% were excluded, (ⅱ) individuals with a call rate less than 95% were discarded, (ⅲ) SNPs with minor allele frequency less than 5% were removed and (ⅳ) the deviated markers (p < 0.1 × 10^−3^) were excluded based on Hardy–Weinberg test. Finally, we performed a relationship test and removed high-related individuals.

### 2.2. Linkage Disequilibrium and Effective Population Size

The effective population size (*Ne*) was defined as the number of individuals that harbor the same distribution of allele frequencies under random genetic drift in an idealized population [21]. In theory, the prediction of *Ne* can be calculated through the linkage disequilibrium (LD) scores. With the availability of high-density chip in pigs, it was feasible to calculate the LD scores between pairwise markers. In this analysis, the LD scores (*r^2^*) between pairwise SNPs was calculated using PLINK v1.07. The effective population sizes (*Ne*) were calculated according to the formula [22,23]:

$$N_{e}=\frac{1}{4c}\left(\frac{1}{E\left(r^{2}\right)}-1\right)$$

where *c* represents the linkage map distance in Morgans [24,25,26]. The physical distance to genetic distance conversion rates were calculated based on total physical chromosome length stated on Sscrofa11.1 (https://www.ncbi.nlm.nih.gov/assembly/GCF_000003025.6) and each chromosome’s genetic length from the National Center for Biotechnology Information (NCBI) (https://www.ncbi.nlm.nih.gov/genome/gdv/?org=sus-scrofa). In addition, *E(r^2^)* was the expected value of *r^2^* for the distance *c*. In addition,

$$t=\frac{1}{2c}$$

where *it* was the time corresponding to *c,* we recruited. In addition, *Ne(t)* reflects the effective population size at *t* generations ago. A series of distance bin between SNPs were given as follows: 0.1, 0.2, 0.5, 1, 2, 5 cM, allowing 5% floating range of SNPs distance, to compute *Ne(t)* before 500, 250, 100, 50, 25, 10 generations. The whole process was performed for SNPs on autosomes [27].

### 2.3. Identification of Runs of Homozygosity

Runs of homozygosity were identified for each available sample separately using PLINK v1.07 which provides a sliding window to detect autozygous fragments [28]. The minimum ROH length was set at 1 Mb to exclude quite common short fragments deriving from LD. To avoid the false discovery rate, the minimum number of SNPs that contained in each ROH was set to 10 which derived from the density of chip. Up to one missing genotype and one heterozygous locus were allowed in a sliding window. In addition, if two SNPs within a segment are too far apart from more than 1 Mb, that segment were split in two [16]. The detected runs of homozygosity were classified by physical length as follows: 1 to <5 MB, 5 to <10 MB and ≥10 MB [7,13]. For each category, the total number of ROH in each chromosome was count for all the available samples.

To explore the distribution of ROH across the whole genome, overlapping regions were obtained from ROH pools with all sampled individuals using PLINK software. To a certain overlapping region, the percentage of individuals containing that region among the whole population was calculated for every overlapping region. The empirical *P*-values were generated by ranking of the percentage values from high to low. Next, we defined core regions as ROH islands in the top 1% of the subjects.

### 2.4. Inbreeding Coefficient

To assess the accuracy of prediction inbreeding effects using ROH, three types of inbreeding coefficients were calculated in this study, including Pedigree-based inbreeding coefficients (*F_PED_*), ROH-based inbreeding coefficients (*F_ROH_*), and inbreeding coefficients based on the excess of homozygosity (*F_HOM_*).

Pedigree-based inbreeding coefficients (*F_PED_*) were calculated for Piétrain pigs, which were born between 2014 and 2017. The pedigree data were recorded by KFNetsKing software. The total record contains 3834 individuals from 126 litters, including 305 experimental samples. They were descended from 34 parent stock (PS) sires and 126 PS sows. Furthermore, there were a total of 29 grandparent (GP) sires, 93 GP sows, 22 great-grandparent (GGP) sires, 59 GGP sows, 10 great-great-grandparent (GGGP) sires and 23 GGGP sows in record. Among 126 litters records, 118 litters have half sibling litter records in this population (Table S1). In order to avoid the inaccuracy caused by the absence of pedigree information, the samples without pedigree records or only having one or two generation records were excluded from analysis sets. Finally, a total of 921 samples were left to calculate *F_PED_* with pedigree depth ranging from 3 to 4 generations. Pedigree-based inbreeding coefficients were estimated by the pedigreemm package in R (https://cran.r-project.org/web/packages/pedigreemm/index.html). Genomic inbreeding coefficients (*F_HOM_*) were calculated for 305 Piétrain pigs with genotype information using the following formula:

$$F_{HOM}=\frac{O_{i}-E_{i}}{L_{i}-E_{i}}$$

In which *O_i_* is the number of observed autosomal homozygous genotypes for individual *i* and *E_i_* is the number of expected autosomal homozygous genotypes for individual *i*, *L_i_* is the total number of genotyped autosomal SNPs. Those parameters were calculated in PLINK v1.07 [28].

Genomic inbreeding coefficient based on ROHs was provided by McQuillan et al. (2008) [29], defined as the length of the autosome covered by ROHs divided by the overall length of the autosome. The formula is:

$$F_{ROH}=\sum L_{ROH}/L_{auto}$$

where *∑L_R__OH_* is the total length of ROHs, and *L_auto_* is the total length of the autosomal genome which is 2478,445 kb in *Sus scrofa* (Pig).

Spearman’s correlation coefficients were calculated between all above inbreeding coefficients through pairwise comparison using SAS.

### 2.5. Functional Annotation for Core Regions of ROH

The core regions of ROH were annotated to identify the known genes in those regions using the genomic database in Ensembl (http://ensemble.org). The core regions of ROH were also matched with previously reported pig Quantitative Trait Locus (QTL) obtained from pig Quantitative Trait Locus Database (Pig QTLdb) (https://www.animalgenome.org/cgi-bin/QTLdb/SS/index). In addition, Gene Ontology (GO) terms and Kyoto Encyclopedia of Genes and Genomes (KEGG) pathways annotations were performed using the list of genes detected in core regions of ROH in The Database for Annotation, Visualization and Integrated Discovery (DAVID) v6.8 tool.

## 3. Results

### 3.1. Characteristics of Genotypic Data

To investigate the pattern of homozygosity, 376 Piétrain pigs were used in chip analysis. After quality control, 4 samples were removed for low genotyping, 1788 SNPs were filtered in missingness test, 2179 markers were excluded based on HWE test and 11,616 SNPs failed in the minor allele frequency test. In addition, we calculated relationship coefficient using identity-by-descent implemented in PLINK v1.07 and 71 samples were excluded (*PI_HAT* ≥ 0.5). Finally, a total of 305 individuals with 35,549 SNPs were available for further analysis.

### 3.2. The Patterns of Linkage Disequilibrium and Past Effective Population Size

**Figure 1:**
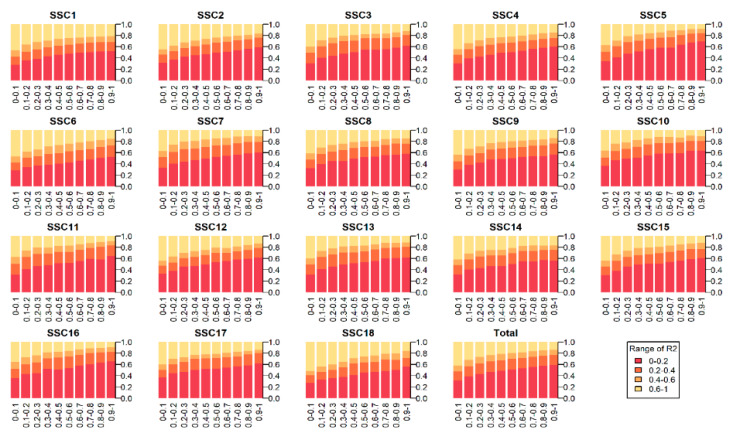
The tendencies of linkage disequilibrium decay across autosomes. The x-axis indicates the diverse classes of distance between SNPs pairs up to 1.0 Mb in each chromosome and the total autosomes, the y-axis indicates the proportion of varied *r^2^* classes.

In this analysis, the LD scores of all pairwise SNPs within a physical distance 10 Mb on the same chromosome were calculated. As shown in Figure 1, the autosomes have similar downward trend. In addition, it can be clearly observed that the mean *r^2^* values decreased gradually with the increase of physical distance.

According to previous studies, the effective population size of commercial pigs has gradually decreased under intensive artificial selection [30,31]. Shin et al. found that the effective population size has reduced by 99.6% through 10,000 generations in Korean Yorkshire [27]. To investigate the tendency in effective population size of Piétrain pigs from 500 generations ago to present, we set a series of bins of interval distances of pairwise SNPs. Then, a vector of LD scores—mean *r*^2^ for each bin could be calculated. Based on the mean *r^2^* values, we estimated effective population size before 500, 250, 100, 50, 25, 10 generations ago. *Ne* at 500 generations ago was about 309 individuals which corresponds to the interval distances of pairwise SNPs about 0.1 cM, and then undergoes a narrowing process. While up to 10 generations ago, *Ne* has reduced to 46 individuals—a reduction of 85.1% (Table 1).

### 3.3. Identification and Classification of ROH

**Figure 2:**
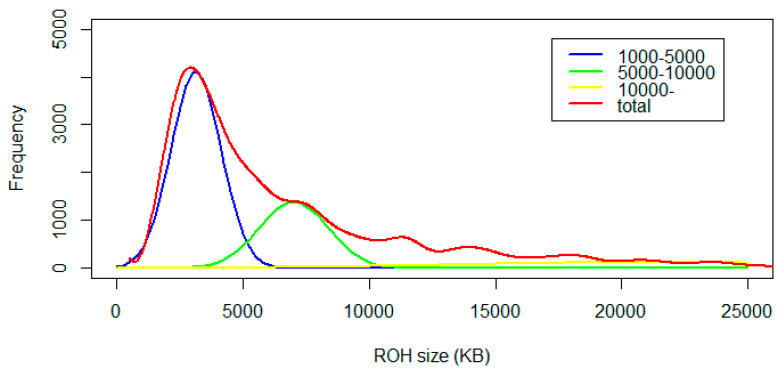
Density estimates of runs of homozygosity (ROH) in three size categories. The x-axis indicates the size of ROH (Kb), the y-axis indicates the value of density.

**Figure 3:**
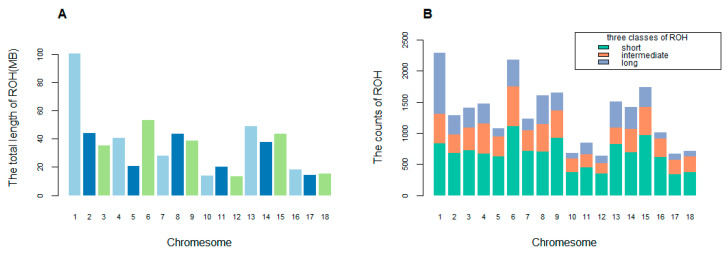
Distribution of ROH in each chromosome. (**A**) Total length of ROH on 18 autosomes. (**B**) Counts of three classes of ROH on 18 autosomes.

In this study, a total of 23,434 ROH regions were identified from 305 available individuals of Piétrain pigs. We found that the total physical length of ROH on 18 autosomes per individual was about 507.267 Mb on average. The density of three size categories ROH was presented in Figure 2. The average length of ROH was 8.195 Mb, and the size of ROH between 1 Mb and 5 Mb accounts for the majority of the total ROH (50.994%). The distribution of total length of ROH in each chromosome was presented in Figure 3A. The total ROH length in chromosome 1 was about 30,553.10 Mb, which was the longest one among all chromosomes. Similarly, the mean length of 13.325 Mb in chromosome 1 was also the largest among all chromosomes. ROHs were classified into three size classes according to the physical length: short (1 to <5 Mb), intermediate (5 to <10 Mb) and long (≥10 Mb). The length of short ROH was 122.216 Mb per individual, the intermediate ROH 144.041 Mb and the long ROH 363.227 Mb. Correspondingly, the proportion of each class to the total number of ROHs was measured: the short ROHs account for 11,950 (50.994%), the intermediate ROHs account for 6269 (26.752%) and the long ROHs account for 5215 (22.254%). The distribution of ROH regions on autosomes was shown in Figure 3B. The greatest number of ROHs across genome was located in chromosome 1 with 2293 regions.

In this analysis, 1574 overlapping genomic regions were obtained from ROH pools. Moreover, 16 ROH regions whose coverage rate are in the top 1% were selected as the core regions. Among them, there are 6 regions in chromosome 1, 5 regions in chromosome 8 and 3 regions in chromosome 6, while chromosome 15 and chromosome 18 harbored 1 region, respectively.

**Figure 4:**
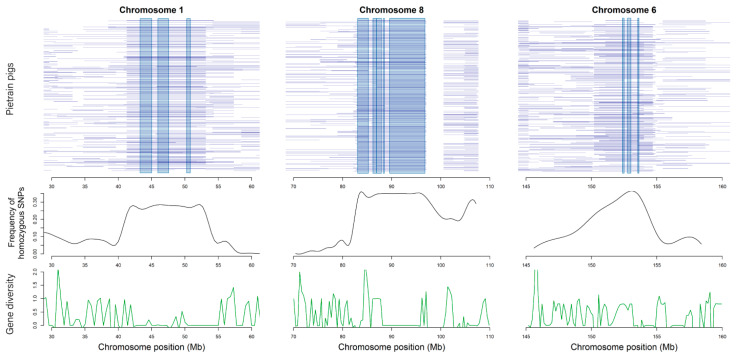
The distribution of ROH core regions in chromosome 1, 8 and 6, the allele frequency and gene diversity in each SNP among the corresponding regions. Blue horizontal lines represent distribution of ROH of per individual. Black line represents frequency of homozygous SNPs averaged in each step of 100Kb. Green line represents gene diversity per SNP.

To further investigate these core regions, we highlighted the distribution of ROHs frequencies, allele frequencies and the gene diversity in Figure 4. For each 100Kb window, we calculated the number of individuals that were homozygous in the window and plot the distribution of homozygous SNPs frequency in chromosomes 1, 6 and 8 by black line. The distribution of the core regions was consistent with the corresponding allele frequencies. Compared with other genomic regions, there were a smaller number of genes in these regions. However, we observed several genes which were related to Piétrain specific economic traits around ROH regions, such as *MRAP2* which was related to energy homeostasis machinery, and *CLGN* which was involved in the control of spermatogenesis and infertility.

### 3.4. Comparison of Different Inbreeding Coefficients

Pedigree-based inbreeding coefficients (*F_PED_*) were estimated using 921 individuals with pedigree depth ranging from 3 to 4 generations. In addition, the genomic inbreeding coefficients (*F_HOM_* and *F_ROH_*) were calculated using 305 individuals with genotype data. The mean value of *F_ROH_* based on the total length of ROH was 0.205. More specifically, the mean of inbreeding coefficient based on short ROH (1 to <5 MB) was 0.049, the mean based on the intermediate ROH (5 to <10 MB) was 0.058 and long ROH (≥10 MB) 0.147.

**Figure 5:**
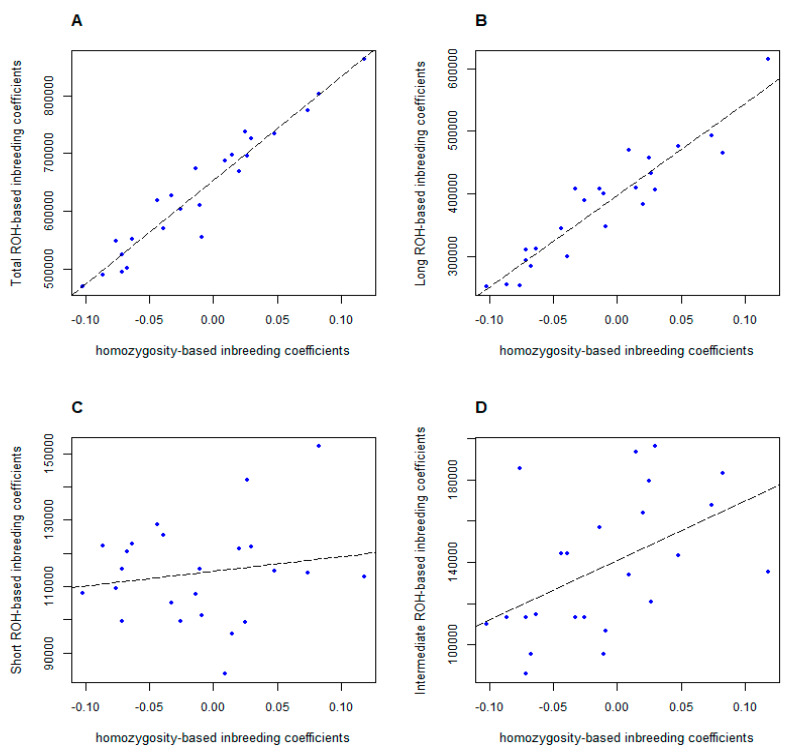
Relationship between ROH-based inbreeding coefficients and homozygosity-based inbreeding coefficients. (**A**) shows *F_HOM_* and *F_ROH_* (total) had a high correlation (*r* = 0.949, *p-value* = 1.908e-06). (**B**) shows *F_ROH_* (long ROH) also was strongly correlated with *F_HOM_* (*r* = 0.910, *p-value* = 2.614e-06). (**C**) shows *F_HOM_* and *F_ROH_* (short) had a low correlation (*r* = 0.038, *p-value* = 0.860). (**D**) shows *F_HOM_* and *F_ROH_* (intermediate) had a low correlation (*r* = 0.499, *p-value* = 0.014).

To further investigate the inbreeding coefficients obtained by different calculation methods, we performed pairwise comparisons between *F_PED_*, *F_HOM_* and *F_ROH_* in samples with both pedigree information and chip data. Among them, *F_HOM_* and *F_ROH_* had a high correlation of 0.949 (*p-value* = 1.908e-06) as shown in Figure 5A, whereas neither *F_HOM_* nor *F_ROH_* was highly correlated with *F_PED_*: *r* = 0.161 (*p-value* = 0.452) between *F_PED_* and *F_HOM_*, *r* = 0.197 (*p-value* = 0.357) between *F_PED_* and *F_ROH_*, respectively (Figure S1). These results suggested computational methods and principles applied in estimating pedigree-based inbreeding coefficients and genomic inbreeding coefficients were distinctive, which also affected the accuracy of calculation, especially in the absence or lack of pedigree information. More specifically, we expected the different categories of ROHs may reflect different population demography. According to previous studies, long ROH was related to more recent parental relatedness, which can reflect the degree of inbreeding effect [8,11]. To confirm this assumption, we have investigated the relationship between the inbreeding coefficients based on three categories of ROHs and *F_HOM_*. The results presented in Figure 5B were consistent with previous assumption, the genomic inbreeding coefficients based on the long ROH was strongly correlated with *F_HOM_* (*r* = 0.910, *p-value* = 2.614e-06). By contrast, neither short ROH nor intermediate ROH has high correlation with *F_HOM_* (*r* = 0.038 for short ROH with *p-value* = 0.860 and *r* = 0.499 for intermediate ROH with *p-value* = 0.014, respectively), as shown in Figure 5C,D. The results suggested that long ROH may arise from recent inbreeding, whereas short and intermediate ROH have different mechanisms.

### 3.5. Functional Annotation for Core Regions of ROH

In this analysis, a total of 16 core ROH regions were investigated and sought for QTLs related to important production traits in pig QTLdb (Table 2). To further investigate the potential biologic significance of those core regions of ROH, the genes that located in the core regions and in the 500 Kb windows around the ROH were annotated. The window size was determined by the LD decay. There were 127 genes around core ROH regions (Table S2). Among those genes, we noticed some of them were related to biologic processes and some of them were even associated with economical traits, including *SLC35F1*, *B3GAT2*, *DCBLD1*, *LMBRD1*, *OMA1*, *MRAP2* and *CLGN* (Table 3).

## 4. Discussion

Runs of homozygosity are contiguous homozygous genotype fragments in the genome that are present in an individual since the identical gametes are inherited from the parents. In previous studies, Bosse et al. studied genetic patterns of ROH from several breeds (n = 52) through 60K SNPchip and the smallest population (Hampshire) only contained 2 individuals [10]. Purfield et al. used multiple breeds of cattle to determine diversity of ROH, including Angus (n = 39), Belgian Blue (n = 38), Hereford (n = 40) and Simmental (n = 58) using 50K SNP [13]. It was reliable that using a larger sample size to investigate the genomic characterization in Piétrain pigs in this study. Overall, runs of homozygosity are widespread in Piétrain genome, but there was no predictable position distribution. All detected ROH were divided by physical length into three categories and the short ROH which accounted for the largest proportion (50.994%) was considered to be caused by LD [39]. We estimated *r^2^* at various distances to represent LD degree. The mean value of *r^2^* calculated for adjacent SNPs in this analysis was 0.557 which was similar to the result that Stratz et al. (2014) estimated in Piétrain pigs [40]. In addition, based on the value of *r^2^*, the mean LD extent can be extended to a distance of 5 Mb, suggesting that LD can help form patterns of the short ROH. As for intermediate and long ROHs that exceed the range of LD extent, they should were broken in the process of recombination, but did not, suggesting that they are supposed to be shaped by other factors [4]. To some extent, the factors like founder effects, genetic drift further increase the difficulty of exploring the ROH formation mechanism.

In population genetics, the decline rate of linkage disequilibrium and the linkage map distance can be used to infer population history and recent effective population size [41]. First, we calculate *Ne* under the assumption that 1 cM approximate to 1 Mb in physical distance following the previous study [31], and the result under this approximation was show in Table S3. In theory, this assumption may bias the value of *Ne* and affect the estimation accuracy, because different chromosomes have diverse linkage conditions due to their distinct physical and genetic distance. Corbin et al. proposed that the assumption that 1 cM approximate to 1 Mb in physical distance would result in lack of consistency for *Ne* estimated [41]. Therefore, the physical distance to genetic distance conversion rates were recalculated based on physical chromosome length and chromosome’s genetic length in this study. We found that each chromosome possesses specific linkage condition, while the decline tendency was identical. In addition, Corbin et al. presented that estimation of *Ne* would be very sensitive and the result would be unreliable when generations <7. Hence, our estimation of *Ne* process terminated at 10 generations. We observed that *Ne* decreased gradually over generations, implying the decline of genetic variations in the long-term evolutionary process. Combining with the prevalence of ROH and numerous short ROH in this analysis, it suggested that the decrease of effective population size, as a signal of decline in genetic diversity, can also reflect high levels of homozygosity indirectly.

It is universally considered that inbreeding was an important factor affecting the pattern of homozygosity. In conventional breeding without available marker data, the inbreeding coefficient, *F*, was estimated through pedigree record, following a principle that an individual transmits an allele to an offspring with 50% probability all the time. However, in the transport process, meiosis was full of randomness with a large stochastic variance. Furthermore, in realistic breeding, it’s common that pedigree record was incomplete or missing. Therefore, it was difficult to trace back the relationship between ancestors and distant parents, which has also become a vital factor affecting the accuracy of inbreeding estimate. This inaccuracy led to the weak correlation between pedigree-based inbreeding coefficients and genomic inbreeding coefficients as we observed.

In recent years, the inbreeding coefficient based on ROH has received more attention, and it was considered to be an accurate estimator to control the probability of inbreeding [5,42]. Compared with *F_ROH_*, another genomic inbreeding estimator *F_HOM_* takes all of the homozygote into account, including separate single locus and homozygous regions. In that case, it will confound all of the history events in experimental population. At the same time, it will increase errors and biases caused by homozygotes generated by random recombination. On the contrary, *F_ROH_* could exclude accidental errors though removing single homozygous and quite short segments. In addition, it has been used in animal genetic analysis [7]. For example, Zhang et al. employed ROH to calculated inbreeding coefficients in cattle population and investigated the distribution of functional variants [11]. Bosse et al. remarked on the impact of demography and recombination on the formation of ROH to master genome inbreeding [10]. Mastrangelo et al. utilized *F_ROH_* to improve mating systems in Italian sheep breeds [6]. Furthermore, based on several recent researches, the short ROH and intermediate ROH were more potentially result from LD and ancient inbreeding, whereas long ROH could represent the level of recent inbreeding more appropriately by excluding obstacle of LD and random effects [8,43,44]. However, the size of boundary between classes was specific to different populations and breeds. Thus, it is more feasible to use total ROH instead of long ROH to estimate level of inbreeding.

In this study, we identified 16 ROH core regions where homozygous rate are pretty high for a specific population, many previous studies revealed that those homozygous segments had a major impact on gene mapping and disease [45,46,47]. Through sequence alignment in Pig QTLdb and gene annotation, we noticed that most of the segments harbored genes which could regulate important biologic functions of Piétrain and most of them may have an impact in economic traits. In this analysis, those regions were mostly linked with meat and carcass traits in Pig QTLdb, for example, *QTL:22,293* harbored in one core region of ROH was related with average backfat thickness. In addition, a great part of them was relevant to production traits, like *QTL:27977*, a location associated with feed conversion ratio. In addition, there were several QTLs associated with reproduction traits, such as *QTL:18325*, a location expression in sperm motility. In addition, some of the core ROH regions also harbored QTLs related to exterior, like *QTL:32743*, associated with breed’s coat color. Among them, we noticed several functional related genes in basic biologic processes, such as *SLC35F1*, a gene played a key role in controlling heat rate; *B3GAT2*, was involved in cellular migration and adhesion in the nervous system; *DCBLD1*, *LMBRD1* and *OMA1*, all participated in compound integral component of membrane. In addition, we also noticed some genes which are related with Piétrain economical traits: *MRAP2* can regulate the energy homeostasis machinery; *CLGN*, an important modulator in spermatogenesis and infertility. As terminal sires in pig industry, Piétrain pig was subjected to high-intensity artificial selection for a long time. Moreover, these observations are consistent with the expectation that homozygosity is associated with some biologic function and important economic traits affected by positive selection. After long-term positive selection, especially more recent artificial selection, regulatory regions that control the important economic traits would be highly homogenous because of directional selection. In other words, those specific regions are easier to be exposed in ROH core regions. Those observed phenomena indicate that positive selection is a significant force shaping patterns of homozygosity in the process of evolution, and the high-frequency ROH can contribute to exploit potential target genes of human-imposed selection.

## 5. Conclusions

In summary, this study investigated the patterns of runs of homozygosity, LD, effective population size and inbreeding coefficients, which provided a clear understanding of how diversity evolved in Piétrain pigs. The decrease of effective population size can mirror artificial selection in breeding process and the recession of genetic diversity. This phenomenon also proves the formation of ROH. Moreover, several genes associated with production traits were found overlapping with ROH regions. The high relationship between inbreeding coefficients (*F_HOM_*) based on homozygous genotypes and the inbreeding coefficients (*F_RO__H_*) based on ROH exposes that the proportion of ROH has an instruction sense in assessing the population’s level of inbreeding. Thus, using genomic information to detect ROH is a powerful method for estimating population inbreeding, especially in assessing the level of inbreeding where pedigrees records were incomplete or missing. Furthermore, the categories of ROHs may be related with the generations on the time frame, short ROH more reflects ancient inbreeding, while long ROH involves in recent inbreeding. Overall, the investigation of ROH patterns provides a helpful reference for selection and assortative mating in modern inbreeding and points an original direction for research of population genetic structure.

## Figures and Tables

**Table 1:** The effective population size over generations for Piétrain pigs estimated from linkage disequilibrium data.

Chr.	cM/Mb	linkage Disequilibrium (r^2^) of Different Interval Distance (Kb)						Estimated Ne in n Generations Ago					
		100	200	500	1000	2000	5000	500	250	100	50	25	10
1	0.5365	0.4472	0.3956	0.3188	0.2413	0.1666	0.0932	576.1	356.0	199.1	146.5	116.5	90.7
2	0.8691	0.4464	0.3976	0.3210	0.2474	0.1721	0.0977	356.8	218.0	121.7	87.5	69.2	53.1
3	0.9723	0.4486	0.4018	0.3248	0.2493	0.1752	0.1000	316.1	191.4	106.9	77.4	60.5	46.3
4	1.0000	0.4482	0.3980	0.3229	0.2488	0.1753	0.1004	307.8	189.1	104.8	75.5	58.8	44.8
5	1.4524	0.4518	0.4019	0.3264	0.2517	0.1766	0.1014	208.9	128.1	71.0	51.2	40.1	30.5
6	0.9690	0.4477	0.3960	0.3204	0.2457	0.1718	0.0989	318.3	196.7	109.4	79.2	62.2	47.0
7	1.2869	0.4495	0.4018	0.3253	0.2495	0.1749	0.1006	238.0	144.6	80.6	58.4	45.8	34.7
8	0.9403	0.4480	0.3999	0.3239	0.2475	0.1731	0.0991	327.7	199.5	111.0	80.9	63.5	48.3
9	0.9893	0.4462	0.3989	0.3238	0.2476	0.1747	0.1006	313.6	190.4	105.5	76.8	59.7	45.2
10	1.8551	0.4508	0.4012	0.3249	0.2490	0.1755	0.1010	164.2	100.6	56.0	40.6	31.7	24.0
11	1.0747	0.4504	0.4015	0.3248	0.2494	0.1746	0.0999	283.9	173.4	96.7	70.0	55.0	41.9
12	1.8242	0.4482	0.3994	0.3229	0.2486	0.1744	0.1006	168.7	103.1	57.5	41.4	32.4	24.5
13	0.6067	0.4505	0.4031	0.3255	0.2490	0.1742	0.0991	502.6	305.1	170.8	124.3	97.6	74.9
14	0.7331	0.4515	0.3999	0.3217	0.2465	0.1714	0.0982	414.3	255.9	143.8	104.2	82.4	62.6
15	0.9021	0.4470	0.4006	0.3242	0.2485	0.1747	0.1005	342.9	207.4	115.5	83.8	65.5	49.6
16	1.1650	0.4498	0.4015	0.3247	0.2503	0.1758	0.1003	262.5	160.0	89.3	64.3	50.3	38.5
17	1.5397	0.4488	0.3997	0.3234	0.2487	0.1740	0.0998	199.4	121.9	67.9	49.0	38.5	29.3
18	1.1859	0.4476	0.3980	0.3222	0.2476	0.1733	0.1003	260.1	159.5	88.7	64.1	50.3	37.8
Total	1.1057	0.4488	0.3998	0.3234	0.2481	0.1738	0.0995	309.0	188.9	105.4	76.4	60.0	45.8

**Table 2:** A list of quantitative trait locus overlapping with the core runs of homozygosity (ROH) regions in Piétrain pig.

Category	Trait	QTL ID
**Meat and Carcass**	Anatomy	QTL:28022,QTL:8699,QTL:154422,QTL:29582,QTL:10245
	Fat composition	QTL:31377,QTL:31364,QTL:31383,QTL:32083,QTL:18547,QTL:101788,QTL:101825,QTL:31356,QTL:31374,QTL:101552,QTL:18546,QTL:31372,QTL:31361,QTL:31368,QTL:18548,QTL:101865,QTL:31360,QTL:6435,QTL:31369,QTL:18549,QTL:101898,QTL:31370
	fatness	QTL:22293,QTL:139226
	Meat color	QTL:36140,QTL:36138
	pH	QTL:23051,QTL:18667,QTL:5871
	texture	QTL:23050,QTL:7819,QTL:7885,QTL:3864,QTL:23052
	Chemistry	QTL:9042
**Health**	blood parameters	QTL:29669,QTL:29665,QTL:29663,QTL:29668,QTL:29670,QTL:29666,QTL:6549,QTL:6318,QTL:29667
	Immune capacity	QTL:127811
**Exterior**		QTL:124729,QTL:17665,QTL:32743,QTL:16383,QTL:125736,QTL:64738,QTL:64740,QTL:64686
**Production**	feed conversion	QTL:27977
	feed intake	QTL:31324
	Growth	QTL:22971
**Reproduction**	Litter traits	QTL:31861,QTL:122507,QTL:64741,QTL:18327
	Reproductive organs	QTL:18371,QTL:126623,QTL:126620
	Reproductive traits	QTL:7662,QTL:18326,QTL:18329,QTL:18328,QTL:18325

**Table 3:** A list of candidate genes overlapping with the core ROH regions in Piétrain pig.

Chr.	Position (Kb)	Length (Kb)	ROH Mapping Frequency^1^	Genes	Gene Function
**1**	53078–53113;	35.052;	76.38%;	*MRAP2*	regulate the energy homeostasis machinery [32]
**1**	50245–50798;	553.125;	73.14%;	*B3GAT2*	implicated in cellular migration and adhesion in the nervous system [33]
**1**	50245–50798;	553.125;	73.14%;	*LMBRD1*	participated in compound integral component of membrane [34]
**1**	43257–450112;	1754.52;	72.17%;	*SLC35F1*	play count role of control heat rate and cardiovascular disease [35]
**1**	43257–45012;	1754.52;	72.17%;	*DCBLD1*	participated in compound integral component of membrane [36]
**6**	153543–153633;	90.199;	69.90%;	*OMA1*	participated in compound integral component of membrane [37]
**8**	86943–87935; 86188–86896;	991.802; 708.867;	68.28%; 66.67%;	*CLGN*	important modulator in spermatogenesis and infertility [38]

^1^ ROH mapping frequency represents the percentage of individuals containing that region among the whole population.

## Supplementary Materials

The following are available online at https://www.mdpi.com/2073-4425/11/5/577/s1, Figure S1: Relationship between genomic inbreeding coefficients and pedigreed-based inbreeding coefficients; Table S1: Pedigree record in Piétrain pigs, Table S2: A list of 127 genes overlapping with the core runs of homozygosity (ROH) regions in Piétrain pig, Table S3: Effective population size over generations for Piétrain pigs estimated based on the assumption that 1 cM ≈ 1 Mb.
