# Supplementary material for: Genome-Wide Patterns of Homozygosity and Relevant Characterizations on the Population Structure in Piétrain Pigs
Source: Genes (Basel). 2020 May 21;11(5):577. doi: 10.3390/genes11050577 (PMC7291003; doi:10.3390/genes11050577)
Supplement: Supplementary file 1 [file genes-11-00577-s001.zip › genes-743076-supplementary-final/Fig.S1.docx]

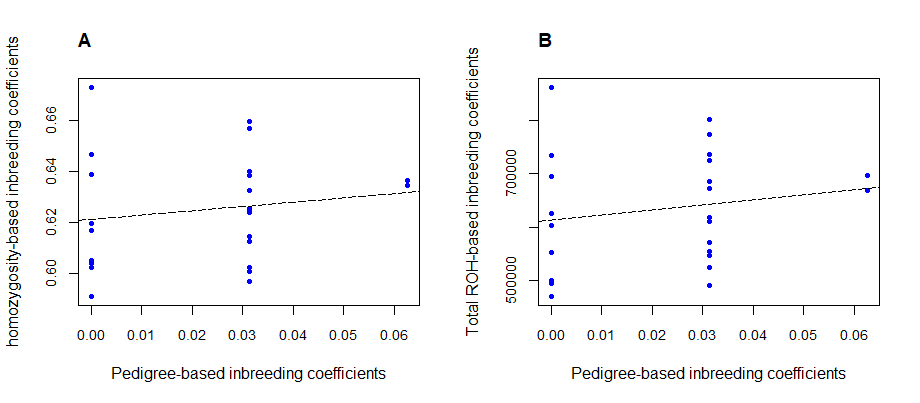


**Figure S1**. Relationship between genomic inbreeding coefficients and pedigreed-based inbreeding coefficients. (A) shows the low correlation between homozygosity-based inbreeding coefficients and pedigreed-based inbreeding coefficients (r = 0.161, p-value=0.452). (B) shows the low correlation between total ROH-based inbreeding coefficients and pedigreed-based inbreeding coefficients (r = 0.197, p-value=0.357).
