# Supplementary material for: Genome-Wide Patterns of Homozygosity and Relevant Characterizations on the Population Structure in Piétrain Pigs
Source: Genes (Basel). 2020 May 21;11(5):577. doi: 10.3390/genes11050577 (PMC7291003; doi:10.3390/genes11050577)
Supplement: Supplementary file 1 [file genes-11-00577-s001.zip › genes-743076-supplementary-final/Table.S2.docx]

Table.S2 A list of 127 genes overlapping with the core runs of homozygosity (ROH) regions in Piétrain pig.

| Chr. | Begin | End | Length（Kb） | ROH mapping frequency | Genes |
| --- | --- | --- | --- | --- | --- |
| 1 | 53078253 | 53113305 | 35.052 | 76.38% | *DPPA5,MRAP2,CEP162,OOEP,CYB5R4,KCNQ5* |
| 1 | 45936751 | 47527329 | 1590.58 | 74.76% | *RF00026* |
| 1 | 51628206 | 51753405 | 125.199 | 73.46% | *RF00026,ssc-mir-30c-2,RIMS1,ssc-mir-30a,RF00156,OGFRL1* |
| 1 | 50244658 | 50797783 | 553.125 | 73.14% | *B3GAT2,FAM135A,SMAP1,LMBRD1* |
| 1 | 43257183 | 45011703 | 1754.52 | 72.17% | *RFX6,ROS1,MAN1A1,PLN,NUS1,VGLL2,GPRC6A,KPNA5,ZUP1,SLC35F1,CEP85L,DCBLD1,PHF3,FAM184A,RF00619,FAM162B,ASF1A,RF00026,PTP4A1,RF00401* |
| 6 | 153542997 | 153633196 | 90.199 | 69.90% | *FGGY,MYSM1,TACSTD2,OMA1* |
| 1 | 208651542 | 213041990 | 4390.45 | 69.26% | *LURAP1L,RF00001,NFIB,TYRP1,MPDZ,RF00100,PTPRD,RF00004,RF00026* |
| 8 | 86943096 | 87934898 | 991.802 | 68.28% | *RAB33B,NDUFC1,ELF2,NOCT,NAA15,MGAT4D,MAML3,TBC1D9,ELMOD2,SETD7,MGARP,SCOC,CLGN,MGST2* |
| 8 | 89592672 | 96804594 | 7211.92 | 67.64% | *SCLT1,RF00015,MFSD8,SLC25A31,PCDH18,RF02216,ABHD18,INTU,JADE1,PABPC4L,HSPA4L,C4orf33,PGRMC2* |
| 6 | 152746637 | 153014393 | 267.756 | 67.64% | *HOOK1,C1orf87,FGGY,CYP2J34,RF00026* |
| 8 | 88277245 | 88526824 | 249.579 | 67.31% | *ELF2,SLC7A11,NOCT* |
| 18 | 25592369 | 25633357 | 40.988 | 66.67% | *CPED1,ING3,TSPAN12,PTPRZ1,WNT16,FAM3C* |
| 8 | 86187542 | 86896409 | 708.867 | 66.67% | *RNF150,CLGN,ELMOD2,MAML3,MGST2,TBC1D9,SCOC,MGAT4D* |
| 8 | 83056568 | 85262039 | 2205.47 | 66.34% | *OTUD4,RF02271,FREM3,INPP4B,ZNF827,RF00001,USP38,IL15,MMAA,GAB1,SMAD1,HHIP,ABCE1,SMARCA5* |
| 6 | 152368013 | 152499472 | 131.459 | 65.70% | *C1orf87,RF00026,HOOK1,FGGY,CYP2J34* |
| 15 | 74717545 | 75245535 | 527.99 | 65.37% | *LRP2,STK39,G6PC2,B3GALT1,SPC25,ABCB11,CERS6,NOSTRIN,DHRS9* |
