# Supplementary material for: Genome-Wide Patterns of Homozygosity and Relevant Characterizations on the Population Structure in Piétrain Pigs
Source: Genes (Basel). 2020 May 21;11(5):577. doi: 10.3390/genes11050577 (PMC7291003; doi:10.3390/genes11050577)
Supplement: Supplementary file 1 [file genes-11-00577-s001.zip › genes-743076-supplementary-final/Table.S3.docx]

Table S3. Effective population size over generations for Piétrain pigs estimated based on the assumption that 1cM≈1Mb.

| Interval Distance（Kb） | Generations ago | linkage disequilibrium (r^2^) | Estimated Ne |
| --- | --- | --- | --- |
| 100 | 500 | 0.4499 | 308.7 |
| 200 | 250 | 0.3963 | 192.9 |
| 500 | 100 | 0.3194 | 107.9 |
| 1000 | 50 | 0.2392 | 80.8 |
| 2000 | 25 | 0.1644 | 65.4 |
| 5000 | 10 | 0.0891 | 54.2 |
| 10000 | 5 | 0.0578 | 45.6 |
